# Early stent fractures in superficial femoral artery resulting multiple pseudoaneurysm formation within one year: a case report

**DOI:** 10.1186/s42155-023-00391-w

**Published:** 2023-09-09

**Authors:** Taylor Benedict, Esraa Hassan, Mikael Mir, Sydney Boike, Jidi Gao, Syed Anjum Khan

**Affiliations:** 1 University of Minnesota Medical School, Minneapolis, MN 55455 USA; 2 Critical Care Medicine, Mayo Clinic Health System, Mankato, MN 56001 USA; 3 Department of Radiology, Mayo Clinic Health System, Mankato, MN 56001 USA

**Keywords:** Superficial femoral artery pseudoaneurysm, Endovascular management, Intravascular ultrasound, Stent fracture, Viabahn covered stent, Everflex stent

## Abstract

**Background:**

Though fracture is known complication of stenting, pseudoaneurysm asscoiated with stent fracture is an extremely rare complication. This has previoulsy been described to occur at least one or more years following initial stent placement. Here we present a case of multi-site stent fracture leading to two separate SFA pseudoaneurysms within one year of placement, successfully treated with covered stents.

**Case presentation:**

A 72-year-old male presented with severe claudication of his left lower extremity (Rutherford 3), found to have long segment SFA chronic total occlusion (CTO). Patient successfully underwent endovascular revascularization. Follow-up duplex ultrasound (US) at one year demonstrated a focus of severe in-stent restenosis (ISR). During repeat angiogram for treatment of the stenosis, stent fracture and pseudoaneurysm was seen in the distal SFA, which was treated successfully with a self-expanding covered stent. Additional stent fractures and pseudoanerusyms were subseuqently identified on follow-up, necessitating a third angiogram, and these were successfully repaired using overlapping covered stents, without further recurrence.

**Conclusions:**

Superficial femoral artery stent fractures leading to pseudoaneurysms are extremely rare, particularly within first year of stent placement. Endovascular repair with covered stents has proven to be an effective treatment option with decreased procedural morbidity compared to surgical repair.

## Background

Peripheral artery disease (PAD) is a pathological accumulation of plaques in the arteries of the extremities and is reported to affect between 4.3% and 5.9% of all individuals aged 40 years or older and 14.5% of all individuals aged 70 and older [10, 15].

Without successful revascularization, peripheral arterial disease can carry significant morbidity, with associated limb loss rate as high as 90% in one year [20]. Treatment has traditionally included surgical bypass, although endovascular treatment has become widely accepted in recent years with similar outcomes for certain patients [20]. Endovascular repair for long-segment occlusion often requires use of stents. Stent fracture in the superficial femoral artery is known to occur in over 10% cases at one year [6, 13, 16]. One extremely rare complication of stent fracture in the superficial femoral artery is the formation of associated pseudoaneurysms. Only a few reports have described this complication, all more than one year after stenting, and treated mainly by open surgical means. Here we describe the first case of multi-site SFA stent fracture with multiple associated pseudoaneurysms, successfully treated by endovascular means.

## Case presentation

**Fig. 1:**
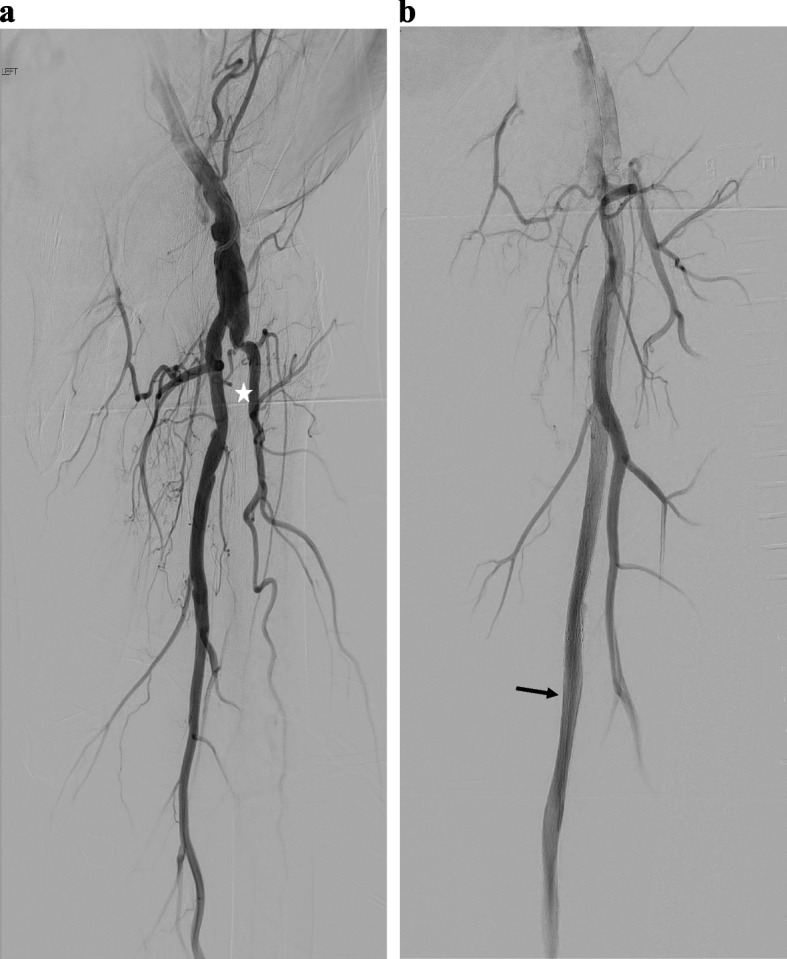
Initial angiogram pre- and post-intervention demonstrating SFA CTO (star) successful recanalization with stenting (arrow) (**a**, **b**)

**Fig. 2:**
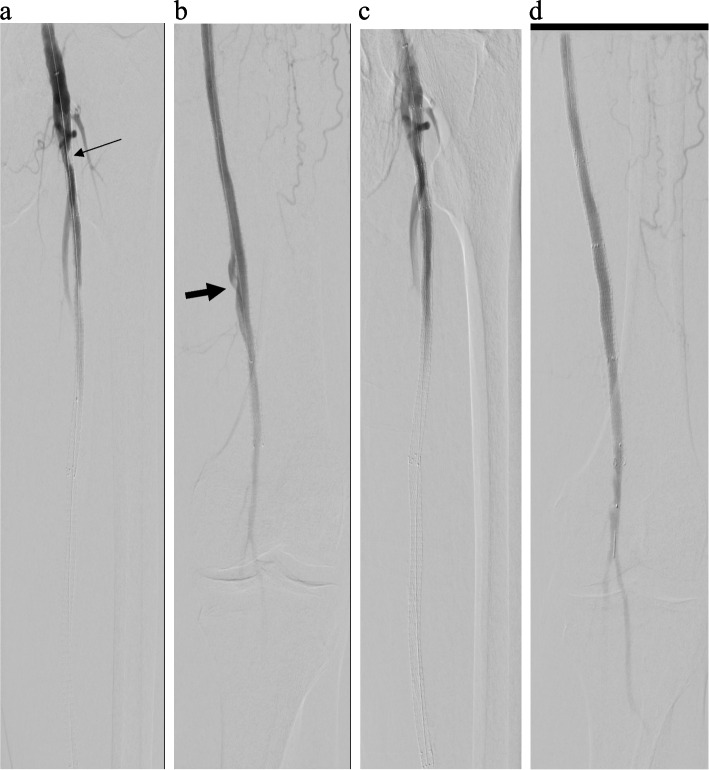
Angiogram 12 months after initial intervention demonstrating focal ISR (thin arrow) in proximal SFA with a distal SFA pseudoaneurysm (thick arrow) (**a**, **b**), treated with angioplasty proximally and Viabahn covered stent placement distally (**c**, **d**)

A 72-year-old male patient with history of stroke, smoking, emphysema, atrial fibrillation, and hyperlipidemia, presented with severe claudication of the left lower extremity (Rutherford 3). Initial arterial duplex US demonstrated diffuse SFA occlusion. The patient underwent a left lower extremity angiogram with recanalization of the long segment left SFA CTO. During the procedure, multiple Everflex stents (Medtronic, Minneapolis MN, USA) were placed, spanning the entire SFA (Fig. 1). Patient was placed on clopidogrel 75 mg daily regimen for 6 months, in addition to his long-term rivaroxiban 20 mg daily regimen. At one year follow-up, the patient continued to show clinical improvement albeit with mild claudication at this point, however repeat ultrasound demonstrated interval development of severe ISR in the proximal left SFA. Repeat angiogram confirmed the finding, which was then treated with angioplasty. During the angiogram, a small distal SFA pseudoaneurysm was identified intraprocedurally. This was successfully excluded by placement of a Viabahn covered stent (W. L. Gore®, Flagstaff, AZ, USA) (Fig. 2).

**Fig. 3:**
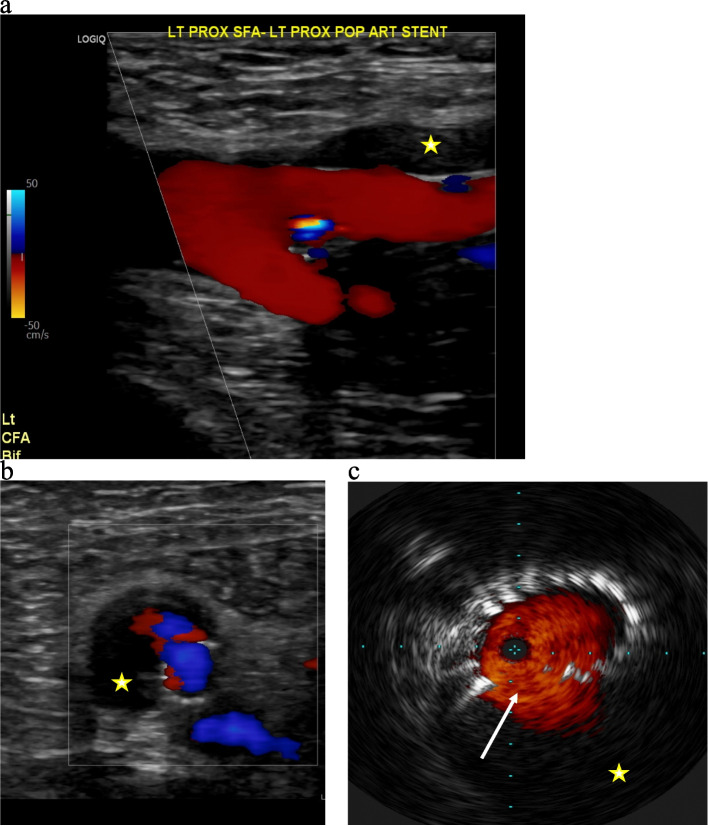
1 month follow-up US following intervention now demonstrating large pseudoaneurysm (star) surrounding the SFA extending to the ostium (**a**, **b**), with corresponding intraprocedural intravascular ultrasound during 3rd angiogram demonstrating large, type II stent fracture (solid arrow) in proximal SFA with flow into large surrounding pseudoaneurysm (**c**)

**Fig. 4:**
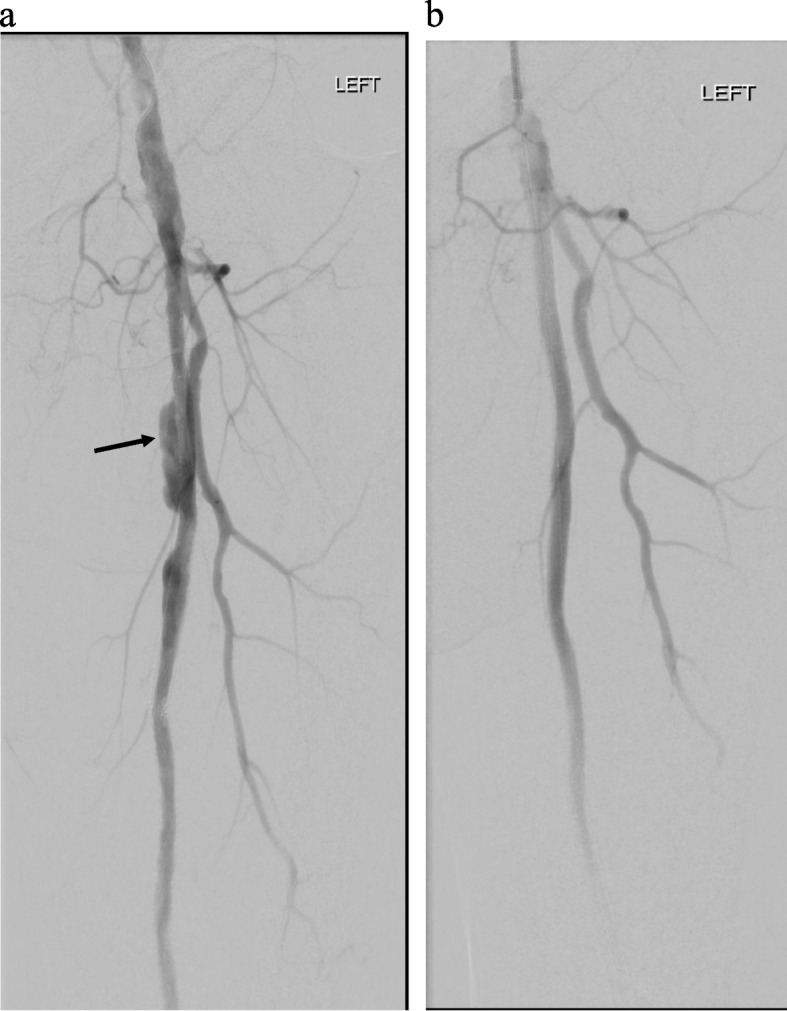
3rd angiogram demonstrating interval development of patent component of proximal SFA pseudoaneurysm (solid arrow) (**a**), successfully treated with placement of Viabahn covered stents to exclude the pseudoaneurysm (**b**)

At 1-month follow-up, patient demonstrated no recurrence of claudication symptoms; however, there was a new SFA pseudoaneurysm on duplex arterial US spanning from proximal to distal SFA, and demonstrating active flow into the pseudoaneurysm sac from the proximal SFA (Fig. 3a). As such, a third angiogram was performed with use of intraprocedural intravascular ultrasound (IVUS), which demonstrated at least two foci of Type II stent fracture in the proximal SFA, resulting in stent deformation and widening at the fracture site, with active flow into the pseudoaneurysm (Fig. 3b). Intraprocedurally, the stent fracture was difficult to identify on angiogram alone, but was much better appreciated on IVUS. The previously repaired site of the pseudoaneurysm remained intact without recurrence. Multiple, overlapping Viabahn covered stents (W. L. Gore®, Flagstaff, AZ, USA) were deployed, from the SFA ostium to the distal SFA (overlapping with the previously placed covered stent), to exclude the flow into the new pseudoaneurysm as well as to prevent future fracture resulting in further pseudoaneurysm formation. Completion angiogram and IVUS demonstrated excellent stent apposition and no further extravasation into the pseudoaneurysm. Fracture of the previously placed bare-metal stents is thought to be the primary cause (Fig. 4). Patient was started on long-term aspirin 81 mg regimen.

At the 9-month follow-up, left SFA pseudoaneurysms remained thrombosed without flow or enlargement, and clinically without signs of recurrence of symptoms of claudication.

## Discussion

 Endovascular revascularization for PAD often entails use of stents with the main objective of restoration and long-term preservation of the patency of the recanalized vessel [7]. In this case, Everflex bare-metal stents (Medtronic, Minneapolis MN, USA) were initially placed in the SFA. Unfortunately, stents placed in the SFA tend to be more prone to fracture or kinking due to movement [17, 19]. Factors that adversely affect long-term patency rate of endovascular repair of SFA include mechanical elongation, compression, and torsion forces placed on the SFA during activities such as ambulation, as well as increased inflammatory response to endovascular treatment compared to other vessels [6].

One specific complication associated with SFA stenting is stent fracture leading to restenosis, late clinical failure, and/or pseudoaneurysm development [13]. The rates of fracture and restenosis differed based upon the type of stent and length of stenosis. The study by Schlager et al. found that 11% of the 220 patients that were stented with either Wallstents (Boston Sci-entific, Natick, MA, USA) (15/78), SMART stents (Cordis, a Johnson and John-son company, Miami Lakes, FL, USA) (8/29), or Dynalink/Absolute stents (Guidant, Santa Clara, CA,USA) (2/113) developed fractures, with the majority of stent fractures being single strut fractures and 28% of that 11% developing a complete circumferential fracture. Another study evaluated the delivery of 56 stents into the SFA and found a 10.7% fracture rate amongst them [16]. However, some sources in the literature are claiming fracture rates as high as 17.8% [2]. The Silveira et al. study also found that the second year after placement was the most common period for stent fracture and was associated with increased length of stenting as well as overlapping stents, accounting for 83.3% of all the fractures identified in this study. This timeline is similar to another study that documented a stent fracture 3 years after placement, leading to the formation of a pseudoaneurysm [1]. Other papers have noted similar timelines for stent fracture (2–3 years), with restenosis rates varying. The Balloon Angioplasty Versus Stenting with Nitinol Stents in the Superficial Femoral Artery (Vienna-ABSOLUTE) trial showed that for mean SFA lesions of 10.1 cm, the rate of restenosis was only 37% at one and two years, and the FAST trial showed that for a mean lesion length of 4.5 cm the rates of restenosis and fracture were 31.7% and 12%, respectively, at 1 year [6].

Stent fractures can be divided into various categories for measurable classification. Various systems exist based on different markers such as number of strut fractures, strut fractures with or without deformity, gaps within the struts, and stent integrity [8, 11, 14, 18]. For example, Goerne et al. divided fractures into four grades: Grade I: single strut fracture, Grade II: two or more strut fractures without deformation, Grade III: two or more fractures with deformation or multiple strut fractures with transection but no gap, and Grade IV: multiple strut fractures with transection and a gap [4]. Another system popularized by Popma et al. divided fractures into two primary groups consisting of isolated strut fractures (further divided into Type 1: single-strut fracture, and Type 2: incomplete transverse fracture) and stent fracture (further divided into Type 3: complete transverse fracture without displacement, and Type 4: transverse fracture with displacement). Regardless of the system, what is most important clinically are the possible complications associated with stent and strut fracture. One such complication being the formation of a pseudoaneurysm.

While SFA stent fractures leading to pseudoaneurysm are extremely rare, they typically occur two or more years after stent placement according to available case reports. In this case, the patient had a multiple overlapping bare-metal stents placed during initial intervention. Within 1 year, the patient was discovered to have two separate sites of wide neck pseudoaneurysm, in the proximal SFA and distal SFA, due to stent fracture. To our knowledge, this is the first case of multifocal fracture leading to multiple pseudoaneurysms of the SFA within a year of stent placement. Previously reported cases followed a similar timeline of events (stent placement, stent fracture, pseudoaneurysm formation), however, they occurred at two, three, and five years [1, 5, 12].

Finally, stent fractures are often difficult to identify on angiographic evaluation alone. IVUS can address many of the limitations of angiography, providing accurate luminal dimensions, visualizations of diseased segments, stenosis, plaque size, stent deployment, and the mechanisms of device failure [3, 9]. In this case, the use of IVUS allowed for much more comprehensive visualization and evaluation of fracture sites, and aided in precise deployment of covered self-expandable stents which were used to maintain arterial flow and exclude the pseudoaneurysm.

## Conclusion

This case demonstrates that while a typical stent fracture leading to pseudoaneurysm formation occurs after two years in the SFA, it can happen within a year of placement. As such, stent fracture should remain a concern even in the early post-operative period, and use of IVUS can help identify and treat this rare, but potentially critical complication. The use of covered stents to reestablish blood flow through the vessel while excluding extravasation or pseudoaneurysm growth has can be an effective endovascular treatment option, minimizing procedural morbidity compared to surgical repair.

## Data Availability

Data sharing is not applicable to this article as no datasets were generated or analyzed during the current study.

## Abbreviations

CTO: Chronic total occlusion
ISR: In-stent restenosis
US: Ultrasound
IVUS: Intravascular ultrasound
PAD: Peripheral artery disease
SFA: Superficial femoral artery
